# Development of a microarray chip for gene expression in rabbit ocular research

**Published:** 2007-02-02

**Authors:** Michael P. Popp, Li Liu, Adrian Timmers, Douglas W. Esson, Lineu Shiroma, Craig Meyers, Scott Berceli, Ming Tao, Graeme Wistow, Gregory S. Schultz, Mark B. Sherwood

**Affiliations:** 1 University of Florida's Interdisciplinary Center for Biotechnology Research, Gainesville, FL; 2 Department of Ophthalmology, University of Florida, Gainesville, FL; 3 University of Florida's Institute for Wound Healing, Gainesville, FL; 4 University of Florida's Vision Research Center, Gainesville, FL; 5 Department of Surgery, University of Florida, Gainesville, FL; 6 National Eye Institute, National Institutes of Health, Bethesda, MD

## Abstract

**Purpose:**

To develop a microarray for the rabbit that can be used for ocular gene expression research.

**Methods:**

Messenger RNA was isolated from anterior segment tissues (cornea, conjunctiva, and iris) and posterior segment tissues (lens, retina, and sclera) of rabbit eyes and used to create two independent cDNA libraries through the NEIBank project. Clones from each of these libraries were sequenced from both the 5' and 3' ends. These sequences and those from the National Center for Biotechnology Information (NCBI) taxonomy database for rabbit were combined and electronically assembled into a set of unique nonoverlapping continuous sequences (contigs). For each contig, a homology search was performed using BLASTX and BLASTN against both the NCBI NR and NT databases to provide gene annotation. Unique contigs were sent to Agilent Technologies, where 60 base oligonucleotide probes were designed and synthesized, in situ, on two different arrays in an 8 array x 1900 element format. Glaucoma filtration surgery was performed on one eye of six rabbits. After 14 days, tissue was harvested from the conjunctiva and Tenon's capsule of both the surgically treated and untreated control eyes. Total RNA from each sample was labeled with cyanine dyes and hybridized to our custom microarrays.

**Results:**

Of the 3,154 total probes present on the two arrays, 2,522 had a signal value above the background. The expression of 315 genes was significantly altered by glaucoma filtration surgery. Genes whose expression was altered included proteins associated with inflammatory response, defense response, and proteins involved in synthesis of the extracellular matrix.

**Conclusions:**

The results of this rabbit microarray study are consistent with those from other wound healing studies, indicating that this array can provide valid information on broad patterns of gene expression. This is the first microarray available for rabbit studies and is a valuable tool that can be used to study molecular events in the eye.

## Introduction

The rabbit, because of its large eyes, ease of handling, and cost effectiveness, has become a standard ophthalmic animal model for many surgical experiments, such as glaucoma filtration surgery (GFS), as well as for the development of new devices and medical therapies. Over the last five years, microarrays, which can simultaneously evaluate changes in gene expression of thousands of genes and are unparalleled in their utility as a discovery tool, have been extensively used to identify the molecular mechanisms of disease. Microarrays are commercially available for the most common animal models. However, there is no microarray for the rabbit. Furthermore, there is little rabbit sequence in public databases from which a microarray could be developed. In this paper we report the sequencing of rabbit tissues and the development of two rabbit microarrays. To test the biological validity of these microarrays as a research tool, we performed GFS on rabbits and compared the results of this study to other GFS and wound-healing studies.

## Methods

### Rabbit eye cDNA library construction

Eight- to nine-week-old, New Zealand white rabbits were obtained from Myrtle's Rabbitry (Thompsons Station, TN). Animals were treated according to the ARVO Statement for the Use of Animals in Ophthalmic and Vision Research and in compliance with institutional animal use and care guidelines. Eyes from two adult rabbits were dissected and separated into two tissue groups: anterior (cornea, conjunctiva, and iris) and posterior (retina and sclera). Two separate cDNA libraries were constructed using a procedure descibed in reference [1]. Briefly, total RNA was extracted from tissue using RNAzol (Tel-Test Inc., Friendswood, TX) and Poly (A)+ RNA was isolated using an oligo-dT cellulose affinity column. An aliquot of the RNA was run on a denaturing gel, and quality was assessed by the presence of a smooth, mRNA-shaped curve of an appropriate range of fragment size. Oligo-dT primed cDNA was synthesized at Bioserve Biotechnology (Laurel, MD) using the Superscript II system (Invitrogen, Carlsbad CA). The cDNA was run over a Sephacryl S-500 HR column (Invitrogen) to fractionate cDNA larger than 500 bp prior to being directionally cloned in *Not*I/*Sal*I sites in the pCMVSPORT6 vector (Invitrogen).

### cDNA sequencing

Methods for sequencing and bioinformatic analysis are described in detail elsewhere [1,2]. Briefly, randomly picked clones were sequenced from the 5' and 3' ends at the NIH Intramural Sequencing Center (NISC). Grouping and identification of sequence tags (GRIST) were used to analyze and assemble the data [2]. Clusters of sequences were also examined using SeqMan II (DNAstar, Madison, WI) to check assembly of clusters and to examine alternative transcripts. Sequences are available through NEIBank.

### Chip fabrication

cDNA sequences from the new rabbit eye libraries were combined with available rabbit sequences from the National Center for Biotechnology Information (NCBI) dbEST database. Paracel Transcript Assembler (PTA; Paracel Inc., Pasadena, CA), which performs a series of sequence cleaning, chimera sequence identification, sequence clustering, and sequence assembly steps, was used to generate a set of nonredundant sequences (contigs) [3]. For each contig, a homology search was performed using the BLASTX and BLASTN application of Paracel BLAST version 1.5.6 (Paracel Inc.) against the NCBI NR and NT databases, respectively. The e-value threshold was set at e-4.

BLAST results were parsed and stored in BlastQuest [4], a SQL database, developed by ICBR that facilitates the management of BLAST results and GeneOntology Consortium (GO) [5] term browsing. AssemblyFilter software, also developed by ICBR, was used to query the top 100 BLAST hits for each contig against the NCBI Gene database, which contains annotation information, including gene function, based on GO terms and metabolic pathway association based on GenMAPP and KEGG pathway database maps [6]. The GO terms and pathway information associated with the lowest e-value and consistent between NR and NT search were assigned to the query assembly. In cases where two contigs mapped to the same gene, the contig assembled from the smaller number of sequences was eliminated to minimize gene redundancy within the entire set of contigs. Finally, AssemblyFilter and ESTScan [7] were used to determine the sequence orientation.

Contigs were submitted to Agilent Technologies (Palo Alto, CA), where a number of probes were designed for each contig with the company's software. Multiple probes were designed for each contig and quality scores, ranging from 1-4, accompanied each designed probe. Due to limited space, we chose to only include one probe per contig and probes with one of the two highest quality scores on the array. Unfortunately, not all the contigs with high quality probes that were designed could fit on the array. All probes with homology to a sequence in the NR database were placed on the arrays. The remainder of the space on the arrays was filled with probes for which we could not find a homologous sequence. Custom arrays were manufactured on glass slides on which 60-base oligonucleotide probes were synthesized in situ with a non-contact printer. Two separate rabbit arrays were manufactured in the eight-pack format (eight individually hybridizable 1.9K arrays per piece of glass). The first set covered 1,577 genes obtained from anterior tissue, while the second set covered 1,577 genes expressed in the posterior tissue. No probe was common to both arrays. Each set of the 1,577 elements included 10 positive and five negative rabbit controls. The remaining elements were Agilent probes for scanner alignment and evaluating dye bias.

### Glaucoma filtration surgery

Six eight- to nine-week-old male New Zealand white rabbits were obtained from Charles River Laboratories (Wilmington, MA). Animals were treated according to the ARVO Statement for the Use of Animals in Ophthalmic and Vision Research and in compliance with institutional animal use and care guidelines. GFS was performed using guidelines described in reference [8]. Briefly, the eyelids were retracted using an eyelid speculum. A partial thickness, corneal traction suture was placed in the superior cornea and used to rotate the eye inferiorly. A limbus-based conjunctival flap was fashioned in the superior lateral quadrant of the eye, approximately 8 mm from the limbus. The conjunctiva and Tenon's capsule were undermined by blunt dissection until the limbus was reached. A clear corneal paracentesis tract was made between the 5 and 7 o'clock positions using a Beaver blade (Becton Dickinson & Co., Franklin Lakes, NJ) and a viscoelastic material (Healon® 10 mg/ml, Pharmacia & Upjohn) was injected to maintain the anterior chamber.

Starting close to the limbus, a needle tract tunnel was created through the sclera and into the anterior chamber using a beveled 22G, IV cannula (Insyte®; Becton Dickinson Vascular Access, Sandy, UT). To reduce the risk of later iris obstruction the cannula was positioned so that its orifice was beyond the pupillary margin, following withdrawal of the metal cannula stylus. The distal end of the cannula was then truncated so that its extrascleral portion was about 1mm in length. The cannula was then tethered to the sclera, to prevent dislodgement, with a single, encircling 10-0 nylon suture (Ethicon Inc., Somerville, NJ). The conjunctiva and Tenon's capsule were finally closed in separate layers, in a watertight fashion, using an 8-0 polygalactan (Vicryl®; Ethicon Inc.) attached to a BV needle and a combined neomycin and dexamethasone ointment instilled into the cul-de-sac.

### RNA isolation and target labeling

Rabbits were sacrificed 14 days after surgery. An approximate 4x4 mm section of bleb tissue, consisting of conjunctiva and Tenon's capsule was harvested, immediately placed in a 1.5 mL microcentrifuge tube, snap frozen in liquid nitrogen, and stored at -80 °C. Conjunctiva and Tenon's capsule tissue from the control eye was harvested, frozen, and stored in the same manner.

Total RNA was extracted from tissue with an RNeasy® Mini column (Qiagen, Valencia, CA). The quality of each sample was evaluated from a 200 ng aliquot with a 2100 Bioanalyzer (Agilent Technologies). Quality was assessed based on the relative abundance of the 18 and 28s ribosomal bands and on the presence of baseline rise, both of which revealed no RNA degradation. A 400 ng aliquot of total RNA was used as template for complementary DNA (cDNA) synthesis with the Low RNA Input Fluorescent Linear Amplification Kit (Agilent Technologies) according to the manufactures protocol. The subsequent cDNA product served as a template for in vitro transcription (IVT), during which one of two cyanine-labeled nucleotides (Perkin Elmer, Wellesley, MA) was incorporated into the synthesized cRNA. All RNA samples from the control eyes were labeled with cyanine 3-CTP (Cy3) while bleb samples were labeled with cyanine 5-CTP (Cy5). IVT reactions were cleaned with RNeasy® Mini columns (Qiagen), and both cRNA concentration and specific activity were measured with a ND-1000 spectrophotometer (NanoDrop Technologies Wilmington, Delaware). The quality of each cRNA sample was evaluated from a 200 ng aliquot with a 2100 Bioanalyzer (Agilent Technologies). Quality was assessed by total yield, specific activity of product, and by the presence of a smooth, mRNA-shaped curve of an appropriate range in fragment size.

### Array hybridization and generation of expression values

Labeled cRNA samples were processed by the University of Florida's Interdisciplinary Center for Biotechnology Research (ICBR) Gene Expression Core Facility (Gainesville, FL). For each rabbit a 100 ng aliquot of the Cy5-labeled sample was combined with a 100 ng aliquot from its paired Cy3-labeled control. This mixture was incubated at 60 °C for 30 min in a high salt buffer to fragment the labeled cRNA into 30-200 base strands. Arrays were hybridized at 60 °C for 17 h in a rotating oven. After hybridization, arrays passed through both a low and high stringency wash according to the manufacture's protocols. Arrays were dried with filtered nitrogen gas and scanned in each of two wavelengths (green: 570, red: 670) with an Agilent G2505 B Scanner (Agilent Technologies). Signal values were corrected for both local background and potential differences in hybridization intensity across the array. A red:green signal ratio was calculated for each element, and ratios were normalized with a lowess transformation. All corrections and transformations of signal values were performed with Feature Extraction software version 8.1 (Agilent Technologies).

A one-group Student's t-test was performed on log2 transformed signal ratios of each probe individually. The null hypothesis, that surgery does not affect probe transcript level, was rejected if the ratio were significantly different from zero. Statistical tests were performed with AnalyzeIt Tools software developed by ICBR.

### Real-time polymerase chain reaction

Quantitative real-time polymerase chain reaction (PCR) was performed on bleb and control RNA samples for five rabbit genes: interleukin 1-β (*IL1β*), matrix metalloproteinase-9 (*MMP9*), transforming growth factor-β1 (*TGFβ1*), transforming growth factor-β2 (*TGFβ2*) and fibronectin (*FN1*). Real-time PCR was simultaneously performed for 18s ribosomal RNA, and its expression served as an internal control. All primers and TaqMan® probes were designed and synthesized by Applied Biosystem (Foster City, CA; Table 1). Reverse transcription was primed with random hexamers. Real-time reactions were performed in a 25 μl volume containing a 1X solution of TaqMan® Universal PCR Master Mix, 200 nM of forward primer, 200 nM of reverse primer, 50 nM of probe, and 20 ng of template cDNA. The reaction was initially heated to 50 °C for 2 min, then the temperature was raised to 95 °C for 10 min, and followed by 40 cycles of 95 °C for 15 s and 60 °C for 1 min. All real-time reactions were performed with the 7900HT sequence detection system (Applied Biosystems, Foster City, CA). Both bleb and control samples were assayed in triplicate. Relative quantitative of expression levels was determined for each gene. All results are expressed as an expression ratio of the bleb to control tissue, normalized against 18s expression levels.

**Table 1:** Primer and TaqMan® probe sequences for selected rabbit genes.

**Gene**	**Forward primer**	**TaqMan probe**	**Reverse primer**
Matrix metalloproteinase-9	CCGGCATTCAGGGAGATG	CTGGGCAAGGGCGTCGTGGTT	TCGGCGTTTCCAAAGTACGT
Interleukin-1 beta	TTGCTGAGCCAGCCTCTCTT	CTGCCATTCAGGCAAGGCCAGC	CTGGGTACCAAGGTTCTTTGAACT
Transforming growth factor beta-2	CGCCAAGGAGGTCTACAAGATAG	CATGCCGTCCTACTTCCCCTCCGA	GGTGGGTGGGATGGCATT
Transforming growth factor beta-1	AAGGGCTACCACGCCAACT	AGTACAGCAAGGTCCTGGCCCTG(7Gs7Cs)	CCGGGTTGTGCTGGTTGT
Fibronectin	GTGGAATACGTGGTCAGTGTCTATG	CCGTTCCGGTTTTGTG	TGGTGGTTACTGCAGTCTGAAC

Nucleotide sequence of the TaqMan® probe and both forward and reverse primers used in real-time PCR reactions. The probe and primer sequences used in real-time PCR reactions and the microarray probe for individual rabbit genes were designed from the same contig.

## Results

### Sequencing

For each library, 3,840 clones were sequenced from both 5' and 3' ends. After removal of empty vector, *E.coli* and mitochondrial contaminants, high quality sequences were obtained for 3,118 and 3,418 clones from the anterior tissue library (library identifier: naf) and posterior tissue library (library identifier: nag), respectively. The average quality read length was 599 bases for naf and 626 bases for nag. After GRIST analysis, the naf data yielded 1,929 potentially unique gene clusters, while nag produced 1870 clusters. Annotated sequence information is available at the NEIBank rabbit library website. In addition, names and annotation of microarray probes for all contigs and those represented on each microarray can be found at the University of Florida Ophthalmic Gene Microarray Project.

### Treatment effects

**Figure 1:**
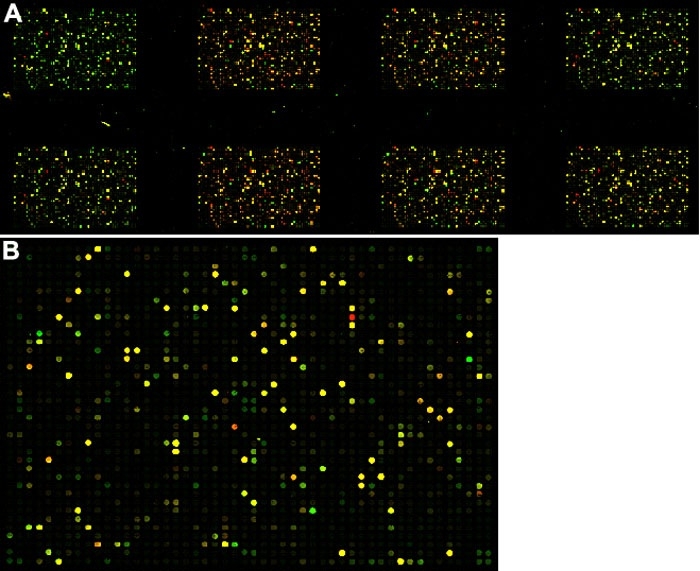
Examples of images generated from scanned microarrays. Example of an image generated from a scanned eight-pack microarray (**A**) and a single 1,900 element (**B**) microarray. Each circle represents a unique spotted probe. Red probes indicate that gene expression in the surgically treated eye is higher than in the control, while green marks higher gene expression in the control. Yellow indicates no difference in gene expression between the surgically treated and control eyes. Black denotes absence of detectable signal.

An example of a scanned slide is shown in Figure 1. Of the 3,154 total probes present on the two arrays, 2,522 had a signal value above the background, in either the red or green channel, on at least two experimental arrays. Genes represented by these probes were considered present. The remainder were considered absent (no expression under any experimental condition). However, some of the absent calls may be attributed to "bad" probes which have suboptimal hybridization characteristics and may not work under any experimental conditions. Only future hybridizations under different experimental conditions will allow us to determine which probes need to be redesigned.

A one-group Student's t-test was performed on log2 transformed signal ratios (red versus green) to identify genes whose expression was significantly effected by GFS. The null hypothesis was rejected if the ratio was significantly different form zero. The expression of 71 and 315 (Table 2) genes was significantly altered by GFS at the p=0.01 and 0.05 level, respectively. The expression of 26 genes changed by more than two-fold and the expression of five by more than four-fold (Table 3). Of the 26 genes that changed by more than two fold, only 16 had an assigned GO annotation for biological process (Table 3). The biological process connected with all but two of these genes is characteristic of those associated with tissue injury and healing.

**Table 2:** Altered gene expression following glaucoma filtration surgery.

**Probe name**	**p value**	**mean log2 difference**	**Hit definition**	**E-value**
UF_Oc_c_40007	0.001	-0.44	13kDa differentiation-associated protein	0.0
UF_Mm_31359	0.023	0.49	2310004I24Rik protein	0.0
UF_Mm_31415	0.022	0.79	2810480G15Rik protein	0.0
UF_Oc_r_30841	0.002	0.36	2-aminomuconic acid semialdehyde dehydrogenase	0.9
UF_Oc_c_40335	0.037	-0.64	3-methyl-2-oxobutanoate dehydrogenase	0.0
UF_Oc_n_41433	0.027	0.51	5-HT3 receptor	0.0
UF_Oc_c_40101	0.044	-0.52	Acetyl-CoA acetyltransferase	0.0
UF_Mm_31324	0.017	0.59	Acid sphingomyelinase-like phosphodiesterase 3a	0.0
UF_Oc_c_40551	0.023	0.33	Adipose differentiation-related protein; adipophilin	0.0
UF_Oc_n_41499	0.008	-0.45	ADP/ATP translocase	0.0
UF_Oc_r_30414	0.022	-0.51	ADP-ribosylation factor-like 6	0.0
UF_Oc_n_41261	0.034	0.30	Aggrecan core protein	0.0
UF_Oc_n_31524	0.005	0.67	Aggrecanase-2	0.0
UF_Oc_r_30810	0.037	0.14	AgrB	0.7
UF_Oc_n_41308	0.020	2.81	Alpha-1-acid glycoprotein	0.0
UF_Oc_c_40520	0.044	0.77	Alpha-1-glycoprotein	0.0
UF_Oc_r_30067	0.004	-1.52	Alpha-actinin-2-associated LIM protein	0.0
UF_Oc_c_40392	0.011	0.92	Anterior gradient 2 homolog	0.0
UF_Oc_c_40535	0.045	0.21	Apolipoprotein D	0.0
UF_Oc_r_30343	0.033	-0.38	ATP synthase, H+ transporting, mitochondrial F1 complex, beta polypeptide	0.0
UF_Oc_r_30203	0.048	0.22	BAP31	0.0
UF_Oc_n_41216	0.007	0.93	Beta casein	0.0
UF_Oc_n_41423	0.010	-0.87	Beta tropomyosin	0.0
UF_Oc_n_41440	0.032	0.57	Beta-arrestin 1	0.0
UF_Oc_r_31084	0.048	0.31	Bifunctional cbiH protein and precorrin-3B C17-methyltransferase	5.0
UF_Oc_c_40296	0.012	-0.12	BMS1-like, ribosome assembly protein	0.0
UF_Oc_c_40651	0.040	0.44	Bromo-adjacent homology domain-containing protein	0.5
UF_Oc_r_31039	0.011	-0.76	C. elegans SRA-13 protein	3.9
UF_Oc_c_40046	0.018	-0.69	Ca2+-transporting ATPase	0.0
UF_Oc_m_40223	0.012	-0.36	Calmodulin 1 (phosphorylase kinase, delta)	0.0
UF_Oc_r_30531	0.003	1.11	Cathepsin B	0.0
UF_Oc_n_41428	0.020	1.05	Cathepsin E	0.0
UF_Oc_c_40013	0.000	2.07	Cathepsin K	0.0
UF_Oc_n_41131	0.030	0.49	CD36 antigen	0.0
UF_Oc_r_31102	0.035	-0.87	Cell cycle control protein	6.0
UF_Oc_c_41026	0.032	0.48	CG11023-PA	7.4
UF_Oc_c_40760	0.024	-0.38	CG6137-PA	1.7
UF_Oc_m_30175	0.013	-0.29	Chaperonin containing TCP1, subunit 2 (beta)	0.0
UF_Oc_m_40241	0.048	-0.29	Chaperonin containing TCP1, subunit 2 (beta)	0.0
UF_Oc_c_40835	0.029	-0.35	Chloroquine resistance marker protein, putative	2.8
UF_Oc_n_41489	0.008	-2.06	Chondromodulin-I	0.0
UF_Oc_y_31574	0.040	0.38	Chromosome 17 open reading frame 37	0.0
UF_Oc_r_30335	0.003	0.52	Complement C4A	0.0
UF_Oc_r_30932	0.018	0.74	Conserved hypothetical protein	1.9
UF_Oc_c_40995	0.013	0.35	Conserved hypothetical protein	6.5
UF_Oc_c_40346	0.040	-1.25	Corneal endothelium specific protein 1	0.0
UF_Oc_c_40478	0.024	-1.66	Corneal endothelium specific protein 1	0.0
UF_Oc_r_30192	0.014	-0.72	CRTAC1-B protein	0.0
UF_Mm_31242	0.027	0.67	Cryptochrome 1	0.0
UF_Oc_n_41539	0.009	1.04	Cystatin B	0.0
UF_Oc_n_41097	0.006	0.47	Cystic fibrosis transmembrane conductance regulator	0.0
UF_Oc_m_40228	0.045	0.25	Cytochrome b	0.0
UF_Oc_r_30452	0.011	-0.32	Cytochrome c oxidase subunit I	0.0
UF_Oc_r_30490	0.003	0.47	Cytochrome c oxidase subunit III	0.0
UF_Oc_n_41352	0.020	-1.35	Cytochrome P450 2A10	0.0
UF_Oc_n_41474	0.024	0.15	Cytochrome P450 8B1	0.0
UF_Oc_r_30439	0.016	0.40	Cytoskeleton-associated protein 1	0.0
UF_Oc_c_40165	0.040	-0.56	DEAD (Asp-Glu-Ala-Asp) box polypeptide 1	0.0
UF_Oc_c_40175	0.004	-0.79	Dihydrolipoamide dehydrogenase	0.0
UF_Oc_r_30308	0.028	-0.72	Dimeric dihydrodiol dehydrogenase	0.0
UF_Oc_r_30024	0.036	-0.46	DKFZP564B167 protein	0.0
UF_Mm_31202	0.004	-0.91	DNA segment, Chr 6, ERATO Doi 253	0.0
UF_Oc_r_30647	0.030	0.33	DNA topoisomerase, type I	0.1
UF_Oc_r_30660	0.039	0.43	DNA-directed RNA polymerase II largest subunit	0.1
UF_Oc_r_30059	0.025	-0.40	DnaJ homolog, subfamily A, member 2	0.0
UF_Oc_r_30305	0.042	-0.23	DSIF p160	0.0
UF_Oc_r_30277	0.002	-0.49	Dynactin 1 0.0	
UF_Oc_m_30346	0.019	-0.82	ENO1 protein 0.	
UF_Oc_r_30721	0.022	0.10	ENSANGP00000019839	0.3
UF_Oc_r_30587	0.016	-0.68	EPAS1 protein	0.0
UF_Oc_c_40498	0.011	0.92	Epithelial chloride channel protein	0.0
UF_Oc_r_30788	0.037	-0.32	Eukaryotic translation initiation factor 2B	0.5
UF_Oc_r_31113	0.022	-0.62	Eukaryotic translation initiation factor 5	6.4
UF_Oc_y_41571	0.006	0.32	EWS/ZSG fusion protein short isoform	0.0
UF_Oc_r_30768	0.014	0.73	Expressed sequence AI462446	0.4
UF_Oc_r_30758	0.028	-0.36	Fibrillar collagen	0.4
UF_Oc_n_31501	0.011	1.28	Fibronectin	0.0
UF_Oc_n_41550	0.002	-0.59	FK506-binding protein 3	0.0
UF_Oc_r_30610	0.017	0.33	Foocen-m2	0.0
UF_Oc_r_30129	0.011	-0.62	FUS/TLS protein	0.0
UF_Oc_r_30813	0.035	0.68	GABA/noradrenaline transporter	0.7
UF_Oc_c_40112	0.050	-0.50	GL004 protein	0.0
UF_Oc_r_30658	0.050	-0.20	GL014	0.1
UF_Oc_c_40973	0.035	-0.78	GLP_680_59866_66603	6.0
UF_Oc_c_40723	0.032	0.97	GLP_82_33920_36064	1.3
UF_Oc_n_31557	0.016	0.77	Glucocorticoid receptor	0.0
UF_Oc_m_30398	0.008	-0.62	Glucocorticoid-induced leucine zipper	0.0
UF_Oc_m_40359	0.016	-0.69	Glucocorticoid-induced leucine zipper	0.0
UF_Mm_31290	0.011	0.27	Glutamine fructose-6-phosphate transaminase 1	0.0
UF_Oc_n_41492	0.033	-0.79	Glyceraldehyde 3-phosphate dehydrogenase	0.0
UF_Oc_n_41246	0.002	-0.80	Glyceraldehyde 3-phosphate dehydrogenase	0.0
UF_Oc_n_41208	0.006	-0.71	Glycogen debranching enzyme	0.0
UF_Oc_n_41424	0.037	0.25	GPI-linked NAD(P+)--arginine ADP-ribosyltransferase 1	0.0
UF_Oc_c_40796	0.047	0.39	Gro-1 Operon gene GOP-1, GOP-1 2.2	
UF_Oc_n_41431	0.016	0.34	GTPase regulator associated with the focal adhesion kinase pp125	0.0
UF_Oc_c_40925	0.019	0.22	Heat shock protein 17.8	4.8
UF_Oc_n_41231	0.050	0.25	Heat shock protein 8	0.0
UF_Oc_c_40953	0.032	0.19	Heme maturase	5.5
UF_Mm_31240	0.022	0.47	Heparan sulfate glucosaminyl N-deacetylase/N-sulfotransferase	0.0
UF_Oc_c_40160	0.000	-0.46	Heterogeneous nuclear ribonucleoprotein C	0.0
UF_Oc_n_41472	0.023	0.21	Histocompatibility antigen DM Heterodimer heavy chain	0.0
UF_Oc_r_30332	0.016	-0.64	HRMT1L2 protein	0.0
UF_Oc_r_30569	0.018	0.33	Hypothetical class II basic helix-loop-helix protein	0.0
UF_Oc_r_30551	0.037	0.74	Hypothetical protein	0.0
UF_Oc_r_30950	0.012	0.69	Hypothetical protein	2.2
UF_Oc_c_40650	0.031	0.65	Hypothetical protein	0.5
UF_Oc_c_40582	0.037	0.49	Hypothetical protein	0.1
UF_Oc_c_40929	0.014	0.49	Hypothetical protein	4.9
UF_Oc_r_30852	0.034	0.42	Hypothetical protein	1.0
UF_Oc_r_31105	0.045	0.38	Hypothetical protein	6.1
UF_Oc_c_40704	0.028	0.25	Hypothetical protein	1.1
UF_Oc_c_40776	0.020	0.24	Hypothetical protein	1.8
UF_Oc_c_41030	0.042	-0.15	Hypothetical protein	7.4
UF_Oc_r_30710	0.049	-0.22	Hypothetical protein	0.2
UF_Oc_c_40581	0.043	-0.37	Hypothetical protein	0.1
UF_Oc_r_30692	0.019	-0.47	Hypothetical protein	0.2
UF_Oc_c_40956	0.006	-0.73	Hypothetical protein	5.6
UF_Oc_c_40928	0.001	-0.75	Hypothetical protein	4.8
UF_Oc_r_30565	0.006	-1.70	Hypothetical protein	0.0
UF_Oc_r_30056	0.043	-0.63	Hypothetical protein BC009732	0.0
UF_Oc_c_41054	0.043	0.45	Hypothetical protein Cj0447	8.0
UF_Oc_r_30796	0.042	0.32	Hypothetical protein Daro140001	0.6
UF_Oc_r_31033	0.005	0.91	Hypothetical protein Daro170901	3.8
UF_Oc_r_30050	0.031	0.52	Hypothetical protein DKFZp761A052.1	0.0
UF_Oc_c_40882	0.025	0.68	Hypothetical protein DKFZp761H221.1	3.5
UF_Oc_r_31126	0.034	0.16	Hypothetical protein Exigu022705	7.3
UF_Oc_c_40979	0.047	0.27	Hypothetical protein FG08398.1	6.2
UF_Oc_c_40111	0.013	0.44	Hypothetical protein MGC14353; thioredoxin (Trx)-related protein, 14 kDa	0.0
UF_Oc_y_31570	0.022	-0.31	Hypothetical protein MGC33212	0.0
UF_Oc_c_40023	0.019	-0.45	Hypothetical protein MGC4825	0.0
UF_Oc_c_40649	0.024	-0.32	Hypothetical protein OB1070	0.5
UF_Mm_31461	0.018	0.42	Hypothetical protein RL076	0.6
UF_Oc_r_30623	0.048	0.32	Hypothetical protein UL126	0.0
UF_Oc_c_40933	0.031	0.29	Hypothetical protein UM01639.1	5.0
UF_Oc_c_40842	0.018	0.42	Hypothetical protein UM01797.1	2.9
UF_Oc_n_41124	0.041	-0.44	Hypoxanthine phosphoribosyltransferase	0.0
UF_Oc_n_41230	0.048	-0.77	Hypoxia inducible factor 1 alpha subunit	0.0
UF_Mm_31343	0.012	0.42	Hypoxia up-regulated 1; calcium binding protein, 140 kDa	0.0
UF_Oc_n_41458	0.016	0.30	Immunoglobulin heavy chain variable region	0.0
UF_Oc_n_41415	0.041	0.41	Immunoglobulin heavy chain VDJ region	0.0
UF_Oc_n_31509	0.046	-0.35	Importin beta-3	0.0
UF_Oc_c_40458	0.017	-0.29	Inositol(myo)-1(or 4)-monophosphatase 1	0.0
UF_Oc_n_31512	0.029	0.59	Integrin beta1	0.0
UF_Oc_n_41542	0.006	0.18	Interleukin 60.0	
UF_Oc_n_41565	0.007	3.11	Interleukin-1 beta	0.0
UF_Oc_r_30860	0.008	0.40	Iron (III) ABC transporter, ATPase component	1.1
UF_Mm_31347	0.033	0.20	Junction adhesion molecule 3	0.0
UF_Mm_31262	0.006	0.30	Karyopherin alpha 2	0.0
UF_Oc_r_30101	0.016	0.38	KIAA0077	0.0
UF_Oc_r_30624	0.044	-0.58	KIAA0653 protein	0.0
UF_Oc_r_30281	0.048	0.25	KIAA0735 protein	0.0
UF_Oc_c_40209	0.001	-0.37	KIAA1289 protein	0.0
UF_Oc_n_41561	0.045	-0.10	Lactase-glycosylceramidase	0.0
UF_Mm_31425	0.011	0.41	Lanosterol synthase	0.0
UF_Oc_c_40685	0.018	-0.56	LD11664p	0.8
UF_Oc_n_41277	0.007	0.60	Low density lipoprotein-related protein 1	0.0
UF_Oc_c_40419	0.042	1.37	Lumican	0.0
UF_Oc_c_40213	0.048	1.05	Lumican	0.0
UF_Oc_c_40194	0.010	1.32	LysozymeC(1,4-beta-N-acetylmuramidase C)	0.0
UF_Oc_r_30224	0.035	0.45	Lysyl oxidase-like protein	0.0
UF_Oc_r_31041	0.034	0.23	Macrolide-efflux protein 3.9	
UF_Oc_c_40724	0.041	0.19	MADS box protein TDR4	1.3
UF_Oc_c_40715	0.038	1.17	Major facilitator family transporter	1.2
UF_Oc_m_40252	0.022	0.25	Mammary tumor integration site 6 oncogene protein	0.0
UF_Oc_n_41311	0.021	0.20	MAPK-activated protein kinase 2	0.0
UF_Oc_n_41197	0.018	0.58	Matrix metalloproteinase-1	0.0
UF_Oc_n_41283	0.008	1.85	Matrix metalloproteinase-9	0.0
UF_Oc_c_40923	0.009	-0.35	Maturase	4.7
UF_Oc_r_30559	0.014	0.24	Membrane protein TGN38 long form	0.0
UF_Oc_n_31527	0.019	0.80	MGC5309 protein	0.0
UF_Oc_c_40323	0.020	0.87	MHC class II histocompatibility antigen RLA-DQ alpha chain	0.0
UF_Oc_c_40271	0.005	-0.50	Mitochondrial ATP synthase, O subunit	0.0
UF_Oc_c_40449	0.021	0.16	Mitochondrial isoleucine tRNA synthetase	0.0
UF_Oc_n_41237	0.006	-1.06	Mono (ADP-ribosyl)transferase	0.0
UF_Oc_n_41350	0.024	0.32	Monocyte differentiation antigen CD14	0.0
UF_Oc_c_40183	0.041	-0.33	Multi-PDZ-domain-containing protein	0.0
UF_Oc_c_40471	0.041	0.78	Mutant alpha-1 collagen type 1	0.0
UF_Oc_c_40493	0.008	0.31	NADH dehydrogenase subunit 3	0.0
UF_Oc_c_40622	0.010	-1.00	NADH dehydrogenase subunit 4	0.3
UF_Mm_31429	0.050	-0.22	NADH dehydrogenase, Fe-S protein 2	0.0
UF_Oc_c_40140	0.004	-0.29	NADH dehydrogenase, Fe-S protein 4	0.0
UF_Oc_c_40409	0.019	-0.70	NADH dehydrogenase1 alpha subcomplex 4	0.0
UF_Oc_n_31543	0.033	0.45	NADP(H)-oxidase	0.0
UF_Mm_31371	0.014	0.14	Nedd4 WW binding protein 4	0.0
UF_Oc_c_40416	0.002	1.83	Neutrophil granules matrix glycoprotein	0.0
UF_Oc_r_30451	0.021	0.32	NIR2	0.0
UF_Oc_n_41335	0.041	0.23	Nitric oxide synthase, inducible	0.0
UF_Oc_r_30229	0.037	-0.55	N-myc downstream-regulated gene 2 isoform b	0.0
UF_Oc_r_30979	0.050	0.40	Non-phototropic hypocotyl 3	2.7
UF_Oc_n_31505	0.013	0.27	Nuclear receptor subfamily 5 group A member 2	0.0
UF_Mm_31360	0.032	0.70	Nudix (nucleoside diphosphate linked moiety X)-type motif 2	0.0
UF_Oc_r_30432	0.046	0.26	OK/SW-CL.33	0.0
UF_Hs_31471	0.011	0.62	Optineurin	0.0
UF_Oc_n_41275	0.038	-0.26	ORM1-like 2	0.0
UF_Oc_c_40096	0.020	0.08	Peptidyl prolyl isomerase H	0.0
UF_Oc_r_30217	0.013	-0.47	Peptidyl-prolyl cis/trans isomerase	0.0
UF_Mm_31468	0.006	0.35	Permeases of the drug/metabolite transporter superfamily	3.9
UF_Oc_n_41377	0.023	-0.76	Phosphatidylethanolamine-binding protein	0.0
UF_Oc_m_30183	0.043	-0.48	Phosphoglycerate kinase 1	0.0
UF_Oc_n_41437	0.030	0.16	Phospholipase A2	0.0
UF_Oc_n_41418	0.018	0.35	Potassium inwardly-rectifying channelJ10	0.0
UF_Oc_m_31072	0.021	-0.53	Prespore-specific protein	4.7
UF_Mm_31207	0.021	0.17	Procollagen, type VI, alpha 3	0.0
UF_Oc_c_40554	0.022	0.40	Proline-rich protein	0.0
UF_Oc_r_30983	0.034	0.30	Prolyl endopeptidase	2.8
UF_Oc_r_30326	0.016	-0.21	Proteasome 26S subunit, non-ATPase, 3	0.0
UF_Mm_31331	0.003	0.63	Protein inhibitor of activated STAT PIASy	0.0
UF_Mm_31303	0.038	-0.56	Protein phosphatase 1D magnesium-dependent, delta isoform	0.0
UF_Oc_c_40192	0.041	-0.40	Protein phosphatase 2, catalytic subunit, alpha isoform	0.0
UF_Oc_c_40376	0.002	0.37	Protein transport protein SEC61 beta subunit	0.0
UF_Oc_r_30690	0.048	0.31	Putative gamma-glutamyltranspeptidase	0.2
UF_Oc_c_40531	0.028	0.72	Putative nuclear protein (1H963)	0.0
UF_Oc_c_40972	0.047	-0.17	Putative signal transducer	6.0
UF_Oc_c_40587	0.011	0.61	Putative yir4 protein	0.1
UF_Oc_c_40146	0.014	-0.56	Pyruvate dehydrogenase E1 component beta subunit	0.0
UF_Oc_r_30286	0.030	-0.40	Pyruvate kinase, M1 isozyme	0.0
UF_Mm_31406	0.007	0.15	Quininoid dihydropteridine reductase	0.0
UF_Oc_r_30365	0.029	0.32	RAB5-interacting protein isoform a	0.0
UF_Oc_c_40043	0.002	0.21	Ribosomal protein L27	0.0
UF_Oc_c_40300	0.042	0.37	Ribosomal protein L31; 60S ribosomal protein L31	0.0
UF_Oc_c_40110	0.038	0.70	Ribosomal protein L5	0.0
UF_Oc_m_30176	0.030	0.39	Ribosomal protein S12	0.0
UF_Oc_c_40373	0.014	0.31	Ribosomal protein S26	0.0
UF_Oc_m_40235	0.003	-0.34	Ribosomal protein S3a; 40S ribosomal protein S3a	0.0
UF_Mm_31352	0.011	0.91	Ribosome binding protein 1 isoform mRRp61	0.0
UF_Oc_c_40391	0.040	-0.33	RIKEN cDNA 1110020P15	0.0
UF_Mm_31177	0.010	0.39	RIKEN cDNA 1300017E09	0.0
UF_Mm_31399	0.017	0.75	RIKEN cDNA 2510025F08	0.0
UF_Mm_31403	0.041	0.16	RIKEN cDNA 4930556P03	0.0
UF_Oc_r_30988	0.039	0.66	RIKEN cDNA 4933437K13	2.9
UF_Oc_r_30495	0.007	-0.76	RIKEN cDNA A530046H20	0.0
UF_Oc_c_40331	0.004	-0.27	Ring-box 1	0.0
UF_Oc_r_30892	0.005	-0.54	RNA polymerase beta chain	1.5
UF_Oc_c_40403	0.050	0.19	SECP43 protein	0.0
UF_Mm_31341	0.008	0.37	Secreted modular calcium-binding protein 2	0.0
UF_Oc_r_30486	0.006	0.38	Seipin	0.0
UF_Oc_n_41531	0.016	1.20	Serum amyloid A-1	0.0
UF_Oc_n_41285	0.001	4.48	Serum amyloid A-3 protein	0.0
UF_Oc_c_40486	0.048	0.36	Similar to 40S ribosomal protein S6	0.0
UF_Oc_c_40398	0.021	0.31	Similar to 60S ribosomal protein L34	0.0
UF_Oc_r_30580	0.028	0.20	Similar to Ac2-210	0.0
UF_Oc_r_30431	0.041	-0.48	Similar to CG5987-PA	0.0
UF_Oc_n_41235	0.012	-0.57	Similar to cyclin I	0.0
UF_Mm_31214	0.044	0.60	Similar to ganglioside-induced differentiation associated protein 3	0.0
UF_Oc_r_30968	0.001	0.28	Similar to hypothetical protein	2.5
UF_Oc_c_40524	0.040	-0.59	Similar to hypothetical protein 9630041N07	0.0
UF_Oc_r_30389	0.012	1.87	Similar to Ldb1a	0.0
UF_Mm_31272	0.038	0.22	Similar to NNX3	0.0
UF_Oc_n_41147	0.008	0.53	Similar to Nucleolar RNA helicase II	0.0
UF_Oc_c_40138	0.044	0.20	Similar to ribosomal protein L30	0.0
UF_Oc_r_30322	0.036	-0.29	Similar to RIKEN cDNA 0610043B10	0.0
UF_Mm_31394	0.034	-0.11	Similar to RIKEN cDNA 5730403E06	0.0
UF_Oc_c_40614	0.038	0.70	Similar to T cell receptor V delta 8	0.3
UF_Oc_c_40202	0.006	-0.51	Similar to ubiquinol-cytochrome c reductase binding protein	0.0
UF_Oc_r_30007	0.039	-0.51	Single-stranded DNA binding protein 2	0.0
UF_Oc_n_41236	0.017	-0.33	SLC26A7	0.0
UF_Mm_31395	0.000	0.87	Sloan-Kettering viral oncogene homolog	0.0
UF_Oc_c_40348	0.030	0.33	Small nuclear ribonucleoprotein polypeptide E	0.0
UF_Mm_31313	0.022	0.68	SMC1 structural maintenance of chromosomes 1-like 1	0.0
UF_Oc_n_41221	0.014	0.44	Sodium/glucose cotransporter 1	0.0
UF_Oc_r_30466	0.030	-0.63	Sodium/potassium/calcium exchanger 3	0.0
UF_Oc_n_31523	0.002	0.44	Sperm-binding glycoprotein ZP2	0.0
UF_Mm_31221	0.001	0.35	Splicing factor, arginine/serine-rich 5	0.0
UF_Oc_n_41289	0.027	-0.59	Succinate dehydrogenase 0.0	
UF_Oc_c_40012	0.047	-0.31	SUMO-1 activating enzyme subunit 2	0.0
UF_Oc_c_40388	0.032	-0.57	Synovial sarcoma translocation gene on chromosome 18-like 2; kiaa-iso protein	0.0
UF_Oc_n_41132	0.049	0.28	T-cell receptor beta-chain precursor	0.0
UF_Mm_31311	0.035	0.49	Telomerase binding protein, p23	0.0
UF_Oc_c_41090	0.038	0.59	Thymosin, beta 4, X chromosome; prothymosin beta 4	0.0
UF_Oc_n_41317	0.014	0.89	Tissue inhibitor of metalloproteinase-2	0.0
UF_Oc_c_41076	0.048	0.59	Transcriptional regulator9.0	
UF_Oc_n_31168	0.016	0.17	Transforming growth factor alpha	0.0
UF_Oc_n_41523	0.020	-0.29	Translation initiation factor eIF-2B-delta	0.0
UF_Oc_c_40042	0.001	0.26	Translokin	0.0
UF_Oc_r_30842	0.002	0.40	Tryptophanyl-tRNA synthetase	0.9
UF_Oc_c_40794	0.035	-0.46	Tubulin beta chain	2.1
UF_Mm_31328	0.020	0.79	Tweety homolog 1	0.0
UF_Oc_r_30020	0.006	-0.70	Ubiquinol-cytochrome c reductase, Rieske iron-sulfur polypeptide 1	0.0
UF_Mm_31210	0.019	0.66	Ubiquitin ligase E3 alpha-II	0.0
UF_Oc_c_40115	0.032	-0.71	Uncharacterized hematopoietic stem/progenitor cells protein MDS029	0.0
UF_Oc_r_30772	0.003	0.76	Unknown	0.5
UF_Oc_r_31071	0.016	0.71	Unknown	4.6
UF_Oc_r_30995	0.023	0.50	Unknown	3.1
UF_Oc_r_30628	0.014	0.44	Unknown	0.0
UF_Oc_c_41047	0.004	0.40	Unknown	7.8
UF_Oc_c_40641	0.017	0.39	Unknown	0.5
UF_Oc_c_41089	0.034	0.30	Unknown	9.8
UF_Oc_m_41084	0.026	0.26	Unknown	9.4
UF_Oc_r_30901	0.037	0.18	Unknown	1.6
UF_Oc_r_30563	0.047	0.12	Unknown	0.0
UF_Oc_r_31141	0.024	-0.14	Unknown	8.0
UF_Oc_c_40874	0.004	-0.23	Unknown	3.4
UF_Oc_c_40868	0.005	-0.37	Unknown	3.3
UF_Oc_r_31012	0.023	-0.39	Unknown	3.5
UF_Oc_r_30622	0.044	-0.41	Unknown	0.0
UF_Oc_c_40813	0.044	-0.47	Unknown	2.5
UF_Oc_c_40821	0.024	-0.60	Unknown	2.6
UF_Oc_c_40282	0.032	-0.82	Unknown	0.0
UF_Oc_c_40775	0.048	-0.94	Unknown	1.8
UF_Oc_c_40341	0.045	0.35	Unnamed protein	0.0
UF_Oc_c_40699	0.021	0.25	Unnamed protein	1.0
UF_Oc_r_30370	0.020	0.20	Unnamed protein	0.0
UF_Oc_c_40420	0.010	-0.45	Unnamed protein	0.0
UF_Oc_m_40305	0.019	-0.50	Unnamed protein	0.0
UF_Oc_m_30347	0.031	-0.61	Unnamed protein	0.0
UF_Oc_r_30055	0.032	-0.71	Unnamed protein	0.0
UF_Oc_r_30237	0.042	0.56	Vacuolar ATP synthase 16 kDa proteolipid subunit	0.0
UF_Oc_n_41218	0.033	0.22	Vacuolar proton-translocating ATPase a2 isoform	0.0
UF_Oc_n_41254	0.023	0.28	Vascular endothelial growth factor receptor 3	0.0
UF_Oc_n_41151	0.008	-0.25	V-erb-a erythroblastic leukemia viral oncogene homolog 4	0.0
UF_Oc_n_41502	0.022	-1.05	Voltage-dependent anion channel 1	0.0
UF_Oc_r_30382	0.003	1.74	X-ray crystal structure of human ceruloplasmin at 3.0 Angstroms	0.0
UF_Oc_c_40769	0.050	0.42	Zn-dependent carboxypeptidase	1.8
UF_Oc_r_31024	0.027	0.49	Zonadhesin	3.6

Genes whose expression was significantly (p less than or equal to 0.05) altered by glaucoma filtration surgery. Mean log2 difference is the level of gene expression in tissue surrounding the surgical site compared to similar tissue from control eye. The p value represents the level of significance in treatment means, the hit definition is the name of the most homologous gene, and E-value is the level of confidence that the matched name is incorrect.

**Table 3:** Genes whose expression was significantly (p less than or equal to 0.05) altered by more than two-fold.

**Probe name**	**p-value**	**Mean log2 difference**	**Hit definition**	**E-value**	**GO biological process term**
UF_Oc_n_41285	0.001	4.48	Serum amyloid A-3 protein	0	Acute-phase response
UF_Oc_n_41565	0.007	3.11	Interleukin-1 beta (IL-1 beta)	0	Antimicrobial humoral response, inflammatory response
UF_Oc_n_41308	0.020	2.81	Alpha-1-acid glycoprotein (Orosomucoid)	0	Acute-phase response, inflammatory response
UF_Oc_c_40013	0.000	2.07	Cathepsin K (EC 3.4.22.-)	0	Lysosome, proteolysis and peptidolysis
UF_Oc_r_30389	0.012	1.87	Similar to Ldb1a	8.46E-37	
UF_Oc_n_41283	0.008	1.85	92 kDa type IV collagenase (Matrix metalloproteinase-9)	0	Collagen catabolism, collagenase activity
UF_Oc_c_40416	0.002	1.83	Neutrophil granules matrix glycoprotein SGP28	1.40E-20	Defense response, innate immune response, cell-cell adhesion
UF_Oc_r_30382	0.003	1.74	Ceruloplasmin At 3.0 angstrom	1.04E-38	oxidoreductase activity
UF_Oc_c_40419	0.042	1.37	Lumican	5.93E-20	Collagen binding, collagen fibril organization
UF_Oc_c_40194	0.010	1.32	Lysozyme C (1,4-beta-N-acetylmuramidase C)	0	Lysozyme activity, response to bacteria, cell wall catabolism
UF_Oc_n_31501	0.011	1.28	Fibronectin	0	Acute phase response, inflammatory response, cell adhesion and migration, extracellular matrix structural constituent
UF_Oc_n_41531	0.016	1.20	Serum amyloid A-1	0	Acute phase response
UF_Oc_c_40715	0.038	1.17	major facilitator family transporter	1.18908	
UF_Oc_r_30531	0.003	1.11	Cathepsin B	3.13E-06	Negative regulation of inflammatory response, proteolysis and peptidolysis
UF_Oc_n_41428	0.020	1.05	Cathepsin E (EC 3.4.23.34)	0	Positive regulation of cytokine secretion, proteolysis and peptidolysis
UF_Oc_c_40213	0.048	1.05	Lumican	0	Collagen binding, collagen fibril organization
UF_Oc_n_41539	0.009	1.04	Cystatin B	0	
UF_Oc_c_40622	0.010	-1.00	NADH dehydrogenase subunit 4	0.335226	
UF_Oc_n_41502	0.022	-1.05	Voltage-dependent anion channel 1	0	Mitochondrial outer membrane
UF_Oc_n_41237	0.006	-1.06	Mono (ADP-ribosyl) transferase	0	
UF_Oc_c_40346	0.040	-1.25	Corneal endothelium specific protein	6.09E-38	
UF_Oc_n_41352	0.020	-1.35	Cytochrome P450 2A10 (CYPIIA10)	0	Electron transport, oxidoreductase activity on paired donors, oxygen binding
UF_Oc_r_30067	0.004	-1.52	Alpha-actinin-2-associated LIM protein	0	
UF_Oc_c_40478	0.024	-1.66	Corneal endothelium specific protein 1	6.32E-09	
UF_Oc_r_30565	0.006	-1.70	Hypothetical protein	0.00204271	
UF_Oc_n_41489	0.008	-2.06	Chondromodulin-I (Leukocytecell-derived chemotaxin 1)	0	Cell differentiation, extracellular space

Mean log2 difference is the level of gene expression in tissue surrounding the surgical site compared to similar tissue from the nonsurgically treated control eye. The p-value represents the level of significance in treatment means, hit definition the name of the most homologous gene and E-value the level of confidence that the matched name is incorrect. Also listed are the GeneOntology Consortium terms for biological process associated with the most homologous gene.

### Real-time polymerase chain reaction

Sufficient quantities of RNA were only available to perform real-time PCR on four of the six original rabbit ocular samples. The changes in expression level of *IL1B*, *MMP9*, *TGFB1*, *TGFB2*, and *FN1* are listed in Table 4. Of these five genes, the expression of two, *IL1B* and *MMP9*, increased dramatically (>100 fold). The expression of two, *FN1* and *TGFB1*, increased moderately (three- to six-fold) and the expression of one, *TGFB2*, was unchanged. The array-based expression changes in *IL1B*, *MMP9*, and *FN1* were generally consistent with those of real-time PCR in that the expression of both *IL1B* and *MMP9* increased significantly and dramatically. For example, of the 3,155 genes present on the array, *IL1B* and *MMP9* had the second and fifth largest change in gene expression, respectively. Additionally, the expression of *FN1* increased significantly, but to a lesser degree than *IL1B* and *MMP9*.

**Table 4:** Real-time PCR results.

**Transforming growth factor beta 2**				
**Sample**	**Green Signal**	**Red Signal**	**Array**	**Real-Time**
1A	73.9**	45.3	-1.38	0.77
2A	21.2	9*	-2.35	0.26
4A	850.6**	83.2	-10.20	0.04
7A	263.9**	43	-96.06	0.15
Mean			-5.00	0.31
**Matrix metalloproteinase 9**				
**Sample**	**Green Signal**	**Red Signal**	**Array**	**Real-Time**
1A	29.7	106.8	3.60	727.2
2A	18.4	158.9	8.64	1928.9
4A	26.2	69.8	2.67	574.4
7A	30.9	110.8	3.59	1020.4
Mean			4.62	1062.7
**Interleukin-1 beta**				
**Sample**	**Green Signal**	**Red Signal**	**Array**	**Real-Time**
1A	14	254.8	18.25	86.03
2A	4.3*	50.4	11.69	258.92
4A	2*	11.1*	5.43	67.75
7A	17.2	306.1	17.82	146.83
Mean			13.3	139.9
**Fibronectin**				
**Sample**	**Green Signal**	**Red Signal**	**Array**	**Real-Time**
1A	9.9*	24.8	2.50	5.68
2A	2.9*	6.1*	2.08	4.65
4A	4.3*	10.2*	2.36	5.36
7A	15.3	29.7	1.94	6.42
Mean			2.22	5.53
**Transforming growth factor beta 1**				
**Sample**	**Green Signal**	**Red Signal**	**Array**	**Real-Time**
1A	8.8	11.5	1.30	2.43
2A	10.3	18.2	1.77	1.64
4A	14.9	12.8	-1.16	1.46
7A	12	11.3	-1.06	6.91
Mean			0.21	3.11

Change in gene expression in tissue surrounding the surgical site compared to similar tissue from the non-surgically treated control eye based on real-time PCR (real-time) and microarrays (Array). Fold change values are arithmetic and not logarithmic. Also listed are the corresponding processed red and green signal values from the microarrays. The asterisk denotes signal values that are indistinguishable from background values and the double asterisk indicates signal values that are inordinately high.

Real-time results for *TGFB1* and *TGFB2* were more difficult to interpret. For *TGFB2*, the signal values in the green channel were inordinately high. Only on the fourth array, where there was a small increase in the red signal, were the real-time and array results consistent. For *TGFB1*, the results were difficult to interpret because all the signal values were close to background levels. On two of the arrays, gene expression goes from present to absent, but the remaining two, gene expression goes from absent to present. Therefore, an accurate estimate of fold change was not possible.

## Discussion

Rabbits, because of their large eyes, ease of handling, and cost effectiveness of their use, have become an important and standard ophthalmic animal model for many surgical experiments as well as for the development of new devices and medical therapies. To date, the research community has been forced to use commercially available chips for similar model systems (rat) due to the absence of rabbit arrays. When this project was initiated, only a small amount of rabbit sequence was available from which microarray probes could be designed. To this end, we have successfully sequenced cDNA inserts from clones originating from rabbit ocular tissues. These sequences are available to the public. In addition, we developed two rabbit arrays containing a total of 3,154 unique contigs.

To the best of our knowledge, this study represents the first report using a rabbit microarray. Because so little rabbit sequence was available prior to this study, it was difficult to make direct comparisons with other rabbit studies, which have focused only on the expression of one or a few genes. Therefore, we compared our results with those from other model systems. Because direct comparisons were made between species and with nonhomologous, studies we compared the general biological interpretation of results between studies.

In a generalized model of wound healing, initial tissue injury stimulates various signaling events including the release of local cytokines and growth factors that eventually led to the production of structural proteins that are components of the extracellular matrix. To evaluate the biological validity of our rabbit microarrays we compared the results with those of our previous rat GFS study [9]. In our rat microarray study, we examined changes in gene expression 2, 5, and 12 days after GFS using the Affymetrix rat 230A GeneChip®. In our current study we took samples only 14 days after surgery. This, in general, mimics the day 12 sample in the rat study. Both the 12- and 14-day sampling times correlate with the latter stages of the wounding response. Therefore, we would not only expect to find similarities between our rabbit 14-day and rat 12-day results, but we would also expect to find gene expression differences characteristic of a late wound stage, principally in genes associated with the structural process of wound healing.

*TGFB2* and connective tissue growth factor (*CTGF*) are two growth factors that mediate the wound-healing process. Their expression generally peaks early in the wound-healing response. For both transcript levels in the rat [9] and protein levels in rabbit [8], *CTGF* was found to reach peak expression levels 5-7 days after surgery. In both cases *CTGF* expression levels returned to presurgical levels by days 12-14. Like the results from the rat microarray, we found no significant difference in *CTGF* expression on day 14 (Table 2). In our rat GFS microarray study, we found the expression level of *TGFB2* decreased 2.5 fold by day 12 [9]. In this study, the *TGFB2* expression level decreased 71%, but this difference was not significant.

In our rat GFS model, scar tissue began to form 7-14 days after GFS [9]. Consistent with this scarring, we saw an increase in the expression of genes which act as structural components of the extracellular matrix. In particular, we found the expression of various collagens, procollagens, biglycan, fibronectin, lumican and vimentin to increase five days after surgery [9]. Expression level of these genes was still elevated on day 12. The results of our current study are, in general, consistent with these results. On day 14, we found both lumican and fibronectin had significantly increased (Table 2). Additionally, lysozyme, a gene thought to be directly involved with the defense against bacteria [10,11], was significantly increased in both the rat and rabbit microarray studies (Table 2). Finally, in our current study *MMP9* was one of the genes that increased the most (Table 3). In the rat microarray study, however, *MMP9* increased only on day 2 and 5 and was back to control levels on day 12 [9]. A number of genes that changed the most on the rabbit array (serum amyloid protein A-1 [*SAA1*], serum amyloid protein A-3 [*SAA3*], cystine-rich secretory protein-3 [*CRISP3*], and alpha-1-acid glycoprotein [*AGP*]) were not present on the rat array, thus, no comparisons could be made with these results.

The acute-phase response is an immediate set of nonspecific host inflammatory responses to tissue injury, surgical trauma, or infection. The response is characterized by the release of proinflammatory cytokines, particularly interleukin-6 (*IL6*), tumor necrosis factor-alpha (*TNFA*), and *IL1B*, which stimulate and mediate the hepatic synthesis and subsequent release of acute-phase proteins (APP) into the blood steam [12-14]. An APP has been defined as one whose plasma concentration changes by at least 25% during inflammation [14]. The concentration of some APP, however, is known to increase by as much as 1,000 fold [13]. The systemic nature of this response constitutes an innate immune response and the APP function to restore homeostasis, neutralize pathogens and promote conditions necessary for tissue repair. APP production in extrahepatic tissues has also been observed in many mammal species and constitutes a local, rather than systemic, response [12-15].

As further biological validation of our rabbit microarrays we would expect to see changes in the expression of genes associated with an acute-phase response, the repair of tissue, and the defense against pathogens. We did find significant changes in the expression of genes involved in the acute-phase response. Of the three primary cytokines that helped mediate this response, only *IL1B, IL6* are represented on our rabbit arrays. The expression of both increased significantly; *ILB1* by 8.6 fold and *IL6* by 13% (Table 2). The gene with the largest increase in expression (22 fold), *SAA3* and its family member, *SAA1*, which increased 2.3 fold, are classified as acute-phase reactants [13] (Table 3). Serum amyloid A consists of a family of apolipoproteins that are highly conserved across all vertebrates and are sensitive markers for inflammation [12,14,16]. *SAA3* is the predominant extrahepatic form in rats, mice, and rabbits [12,17] and was required for effective stimulation of collagenase by *IL1B* in rabbit corneal fibroblasts [18].

The transcript level of three other APP genes, *AGP*, ceruloplasmin (*CP*), and *FN1* was significantly increased following GFS. Both *AGP*, whose expression increased 7 fold, and *CP*, whose expression increased 3.3 fold, are believed to act as antiinflammatory and immunomodulatory agents (Table 3). Therefore, it has been hypothesized that their extrahepatic expression functions to reduce inflammatory induced tissue damage [14,19,20].

*FN1* gene expression increased 2.5 fold following GFS (Table 3). In humans, it appears that a single gene codes for two distinct forms, cellular and plasma. The plasma form is classified as an APP. The cellular form, however, is the major cell surface glycoprotein of many fibroblast cell lines. A major *FN1* function is in the adhesion of cells to extracellular matrix (ECM) materials, particularly collagen, and its presence is therefore instrumental in tissue repair [21]. We observed the transcript expression level of two other ECM-associated genes, lumican (*LUM*) and *MMP9,* to be increased after GFS. *LUM* increased 2.5 fold (Table 3). LUM is present in large quantities in the corneal stroma, where it not only interacts with collagen molecules to limit fibril growth, but also plays a critical role in the regular spacing of fibrils and acquisition of corneal transparency [22]. *MMP9* expression, increased 3.6 fold. *MMP9* is an endoprotease that cleaves matrix substrates, such as gelatin and collagen types IV, V, and VII, and therefore, plays a major role in the alteration of the ECM after tissue injury [23,24]. *MMP9* was shown to be upregulated at both the transcriptional and translational levels by SAA in human THP1 cells [23]. Also of note is that MMP9 was found to be upregulated at both the mRNA and protein level in mononuclear blood cells of normal-tension glaucoma patients [24].

As with the increase in the expression of genes associated with the acute-phase response and tissue repair, we found the expression of defense-related genes to increase. The expression of lysozyme C and *CRISP3*, which are principal enzymes of the innate immune system, increased 2.5- and 3.6 fold, respectively (Table 3). Lysozyme is a protein that degrades bacterial cell walls. It is a component of granules of neutrophils and the major secretory product of macrophages [10,11]. Similarly, *CRISP3*, which was originally purified from human neutrophils, is present in the gelatinase granules of human neutrophils, along with lysozyme, collagenase, and gelatinase [25].

It is clear that our rabbit microarrays are providing gene expression results that are compatible with those found in similar tissue injury, wound healing and GFS surgery studies. For example, the genes that changed the most, *SAA1*, *SAA3 AGP*, and *FN1* are acute-phase reactants. If not directly involved, they are at least markers for inflammation. Other genes showing large changes include *IL1B*, which is known to mediate inflammatory responses, *LUM* and *MMP9*, which are associated with degradation and/or remodeling of the ECM, and both lysozyme and *CRISP3* which are implicated as a direct defense against pathogenic challenge. Our microarray results are also generally consistent with the magnitude and direction of real-time PCR results when microarray signal values are well above background levels.

The availability of rabbit microarrays affords a new and unique opportunity for identifying molecular controls for ocular processes. An addition benefit is that these microarrays have been successfully used for a nonophthalmic study involving vascular tissue (Dr. Scott Berceli, personal communication). Fortunately, relatively few genes exhibit tissue-specific expression, and therefore, fulfill a similar function in different cell types. Thus, gene-expression and associated cellular behavior and responses across multiple tissue types, including epithelial, connective, neurological, and immunologic, may potentially be investigated using this technology, implying a utility for more than ocular research alone. The greatest limitation of these microarrays is that they only represent approximately 3000 unique genes. Further sequencing is underway to obtain a greater coverage of the expressed rabbit genome. This will no doubt, enhance the utility of the rabbit microarrays.
